# Understanding the Development of Elite Parasport Athletes Using a Constraint-Led Approach: Considerations for Coaches and Practitioners

**DOI:** 10.3389/fpsyg.2020.502981

**Published:** 2020-09-30

**Authors:** Nima Dehghansai, Srdjan Lemez, Nick Wattie, Ross A. Pinder, Joe Baker

**Affiliations:** ^1^School of Kinesiology and Health Science, York University, Toronto, ON, Canada; ^2^Department of Kinesiology and Health Promotion, California State Polytechnic University, Pomona, Pomona, CA, United States; ^3^Faculty of Health Sciences, Ontario Tech University, Oshawa, ON, Canada; ^4^Paralympic Innovation, Paralympics Australia, Adelaide, SA, Australia

**Keywords:** expertise, models, theoretical framework, constraint-led approach, coach resource, athlete development, Paralympics, Para sport

## Abstract

For the past half-century, the Paralympic Games has continued to grow, evident through increased participation, media recognition, and rising research focus in Para sport. While the competitive pool of athletes has increased, athlete development models have stayed relatively the same. Currently, coaches rely mainly on experiential knowledge, informal communication with colleagues, and theory transferred from able-bodied contexts as main resources to support development for themselves and their athletes. The purpose of this paper was to introduce Newell’s constraint-led model and its multidimensional spectrum and practical scope to address the complexities of athlete development. The model consists of three overarching constraint categories (i.e., individual, task, and environment) along with proposed additional sub-categories to capture nuances associated in Para sport in order to provide additional context to coaches regarding athlete development. Utilizing this theoretical framework, we present a holistic approach for coaches and practitioners to consider while addressing athletes’ short- and long-term developmental plans. This approach highlights the interactions among factors from a wide range of categories that indirectly and directly impact one another and ultimately influence athletes’ developmental processes. It is important to consider the dynamic interaction of constraints over various timescales during development and identify underlying issues to improve athlete experience and maximize developmental opportunities. Coaches and practitioners can use the proposed framework as a guide to key factors to consider for their cohort of athletes. This approach provides a context-specific approach that considers unique factors associated with athletes and their environment.

## Introduction

The Paralympic Games and the Para sport community have seen tremendous growth with 2.15 million spectators watching 4,328 athletes from 159 countries compete in 22 sports in the most recent Summer Paralympics in Rio de Janeiro, Brazil (Paralympics, 2019). Parallel to this, media recognition along with research in this area, has increased in pace (Houlihan and Chapman, 2017). Given this growing popularity, contextualizing the existing research on athlete development may provide a broader understanding of the factors that influence participation, development, and expertise in Para sport. A notable issue with current models is the aim *and* need to generalize and condense all athletes into one developmental pathway. Such models are considered to be necessary to understanding development; they provide direction and identify specific roles for individuals within the complex sporting structure while providing a framework that organizations can utilize to evaluate and allocate resources and funding. However, the rigidness and need for a “neat and tidy” model ignores the variability that exists in all athlete development trajectories which is exacerbated by numerous factors in Para sport, including disability-related nuances. While models have been examined and publicly scrutinized (Côté, 1999; Lloyd and Oliver, 2012), the underlying motive of these articles has usually been to promote an alternative model. However, the larger issues underlying all models are: classification of athletes into categories, the generalization of the pathway, and time-related (biologically referenced) assumptions to development (Lloyd and Oliver, 2012; MacNamara et al., 2014).

Thus, the purpose of this paper is not to suggest another model, similar to what is currently being applied across the globe [e.g., Long-Term Athlete Development model (LTAD); Balyi and Hamilton, 2004 or Foundation, Talent, Expertise, Mastery model (FTEM); Gulbin et al., 2013], nor is it to propose specific guidelines to change policy [e.g., SPLISS (Patatas et al., 2020)]. The objective of this paper is to provide guidelines for coaches and practitioners to make more informed decisions by understanding the scope of variables that interact to impact athlete development at any given time. We aim to conceptualize current understanding in athletes’ development and provide a better understanding of the dynamic interaction of the myriad factors influencing athletes’ development. However, a limiting factor in this area is the lack of a comprehensive theoretical framework to guide research and applied work; without a theory to guide research and interpret findings, it may be difficult to understand the factors that influence processes and outcomes (Coalter, 2007). Without an overarching framework, it may also be difficult to parse existing research to generate better avenues for future research. In this paper, we demonstrate how Newell’s (Newell, 1986) constraints-based model is a useful framework to (i) organize current literature, (ii) promote a discussion of predominant issues in the development of Para sport athletes, and (iii) identify practical methods to inform coaches/practitioners of factors to consider in athletes’ development. While there has been a growing body of literature in Para sport, very little of this research has considered the dynamic interaction across developmental factors and how each can directly or indirectly impact the behavior and outcome of another. More importantly, there is limited research that has considered how this dynamic interaction takes shape in an applied setting where athletes continuously interact with their environment and take on tasks that require different demands.

## Newell’s Constraints Model

Newell’s model has been used to provide structure to systematic reviews (Rienhoff et al., 2016), highlight biomechanical interactions in Para sport (Keogh, 2011), support skill development in Para sport (Pinder and Renshaw, 2019), and more importantly, as a theoretical model to understand athlete development (Phillips et al., 2010; Renshaw et al., 2012; Wattie et al., 2015). The appeal and longevity of Newell’s model is likely due to the straightforward yet multidimensional categories and the interactive nature of their relationship. Newell’s theoretical framework is also attractive for our purposes because it is consistent with definitions of disability as biopsychosocial phenomena resulting from interactions between individuals and contextual-environmental factors (Leonardi et al., 2006).

**FIGURE 1:**
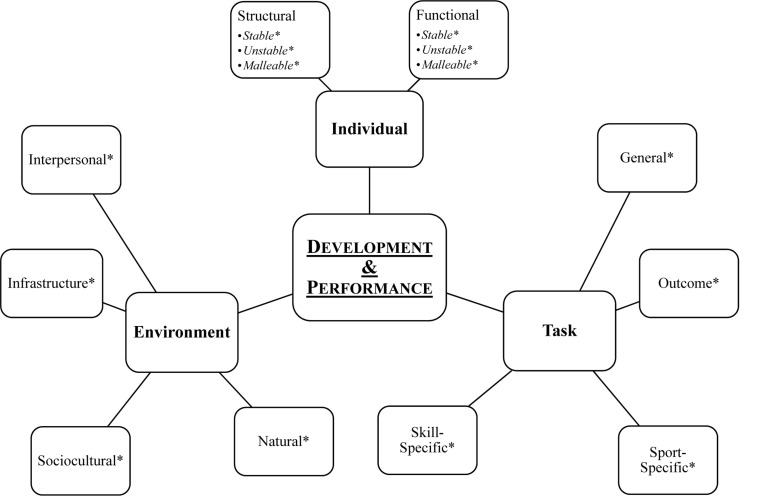
Illustration of Newell’s constraint-led model with the addition of suggested categories. ^∗^ Indicates sub-categories that are proposed to better organize the themes under each tenet.

This model, like many developmental systems theories (Lerner et al., 2005a,b) emphasizes the integration and connectedness of different constraints^1^ that dynamically interact over time to affect developmental outcomes; however, it differs from other models due to its ease of practical application (see Figure 1 for depiction of the model). Often depicted as points on a triangle, Newell’s framework includes task, individual (i.e., performer), and environmental constraints (Newell, 1986, 1991). Changes to any of these constraints, or the interaction between multiple constraints, will modify outcomes. In the following section, we expand on these categories and use Newell’s theoretical framework in combination with the existing literature to discuss how these constraints may influence the development of Para sport athletes and identify how this framework can be utilized to help coaches shape an environment optimizing athletes’ development (see Table 1 for a short description of each type of constraint). The proposed additional constraint sub-categories are drawn from the authors’ expertise both academically and practically working with ecological and constraint-led approaches in able-bodied and Para sport systems. This work sheds light on current gaps and limitations and we aim to address these nuances by specifying additional categories that could help coaches and practitioners better prepare for working in the Para sport context.

**TABLE 1:** Description of each constraint within Newell’s constraint-led model.

	**Constraints which govern the…**	**Example**
**Task**	…parameters of an activity	
General	…process of a task	Ability to control a racquet
Outcome	…results of a specific event	Points scored in a game or outcome of the game
Sport-Specific	…operations of a sport in general	Athletes’ classification
Skill-Specific	…execution of a specific skill	Performing a forehand in wheelchair tennis
	**Constraints which govern individual’s…**	
**Individual**	…capacity of the performer	
Structural Stable	…body structure that are stable over time	Height, weight
Structural Malleable	…body structure that are adaptable to task demands and relatively long-term	Improved cardiovascular performance
Structural Unstable	…body structure that are transient and bidirectional in nature	Soreness, impairment-related day-to-day changes
Functional Stable	…behavior that are stable over time	Personality trait
Functional Malleable	…behavior that are adaptable to task demands	Self-efficacy
Functional Unstable	…behavior that are transient and vary on day-to-day basis	Day-to-day changes, such as mood, arousal, etc.
	**Constraints which govern the…**	
**Environmental**	…conditions of individual’s surrounding	
Natural	…conditions of individual’s habitat	Climate, geographical position
Infrastructure	…physical infrastructures of individual’s surroundings	Accessibility to training centers
Sociocultural	…operation of social structure in individual’s surroundings	Policies, social beliefs
Interpersonal	…interaction between the individual and their social environment	Coaches, teammates, parents, and friends

### Performer Constraints

To better account for the different ways that individual characteristics can influence outcomes, individual constraints in Newell’s model are considered relative to two sub-categories, structural and functional (Newell, 1986, 1991; Haywood and Getchell, 2009). In addition, given some aspects within each sub-category are relatively stable (e.g., height, limb length) while others are more easily changed through training (e.g., physical fitness, weight), we suggest three additional groupings within each sub-category: *stable* (limited to no change over time), *malleable* (changes over medium to long timescales), and *unstable* (random fluctuations over short, medium, and long timescales).

#### Stable Structural Constraints

Stable structural variables (e.g., height) affect performance through their influence on a myriad of variables that need to be optimally coordinated in order to attain a given performance outcome. Differences in height provide players with different performance/action opportunities (often referred to as affordance from an ecological dynamics perspective; Araújo et al., 2009), which may have important consequences for development (e.g., the advantage of seated height (torso length) in wheelchair basketball). In addition, physical limitations as a result of athlete impairment lead to unique action capabilities. For example, wheelchair basketball athletes with more severe impairments may compensate for limitations in trunk muscle activation by increasing the flexion of their shoulders, elbows, and wrists during free-throw shooting which causes more variability in their shot and decreases shooting percentage (Goosey-Tolfrey et al., 2002; Malone et al., 2002). Resultantly, players in different classifications are often assigned different roles to compensate for their physical function (e.g., lower-class players set picks for higher class players who have the ability to maneuver and increase speed during short-distance sprints; Vanlandewijck et al., 2003, 2004; Sporner et al., 2009). Understanding stable structural constraints can provide coaches with the opportunity to adapt their game strategies to utilize each athlete in a unique way to maximize their performance and overall contribution to the team (Boyd et al., 2016; Haydon et al., 2018; Seron et al., 2019). However, stable structural constraints should not be considered as lone functioning constraints that impact athletes’ performance, as they are likely interdependent on malleable structural constraints which are prone to change over time (Marszałek et al., 2018).

#### Malleable Structural Constraints

Consistent with Newell’s reference of time scales for skill acquisition and development, malleable constraints are identified as body functions that adapt to the demands of the task due to extensive training or change due to natural course of progression (Newell et al., 2001, 2009, 2010). While some impairments can cause irreversible damage (e.g., nerve damage in spinal cord injuries, amputation of a limb), some bodily functions that are a symptom of the impairment (e.g., decreased cardiovascular function due to spinal cord injury [SCI] or phantom limb) can change over time (Bläsing et al., 2010). For example, Para sport athletes training long-term have been shown to exhibit superior cardiovascular performance than untrained able-bodied individuals (Huonker et al., 2003). In addition, Para sport air-pistol shooters display higher alpha level activities in the frontal, central, and temporal regions during shooting performance, highlighting the neuroplasticity of the brain and ability to recover from earlier injury to demonstrate greater attentional demand when executing a visual task (Kim and Woo, 2013). As such, it is important to consider *how* physical capabilities can change over time based on specific types of training and consider how this may impede or facilitate skill acquisition. The ability to differentiate athlete capabilities that are influenced by impairment versus acquired skill has been a long-standing challenge for classifiers and practitioners (Beckman and Tweedy, 2009; Tweedy and Vanlandewijck, 2011; Vanlandewijck et al., 2011). While malleable constraints capture nuances associated with long-term change, unstable structural constraints are, as Newell et al. (2001, 2009, 2010) suggest, “transient,” and change more frequently in shorter periods of time.

#### Unstable Structural Constraints

Unstable structural constraints are physical and physiological factors that can vary unpredictably on a day-to-day basis. For example, while an athlete may demonstrate improvements in skill execution across multiple training sessions, there may be variability between practices that is influenced by their physical or psychological well-being. This can be due to random factors such as sickness or more systematic factors such as stiffness, soreness, and/or impairment-related complications such as day-to-day variabilities associated with conditions such as Multiple Sclerosis or Cerebral Palsy (Hertel, 2002; Meberg and Broch, 2004; Barkoudah and Glader, 2018). Therefore, it is vital for coaches to be flexible and understand the variability associated with athlete development. In particular, coaches and researchers should consider that these constraints are bidirectional and unstable, and focus should be on the long-term trajectory while negotiating minor setbacks.

#### Stable Functional Constraints

Stable functional constraints relate to internal factors such as personality traits, which are generally stable over time. Cox and Davis (1992) compared the personality traits (anxiety control, concentration, confidence, mental preparation, and motivation) of Paralympic athletes to athletes from able-bodied sport and results indicated higher positive scores for Paralympians on anxiety control, confidence, and motivation. This finding was subsequently supported by Patten et al. (1994), who using a different sample found similar scores in iceberg mood profiles (i.e., T scores below the 50th percentile on Tension, Depression, Anger, Fatigue, and Confusion and above the 50th percentile on Vigor). In addition, Pensgaard et al. (1999) compared Paralympic and Olympic athletes’ motivational climate under the achievement goal theory. Athlete profiles were reported to be relatively similar: ego and task orientation levels were similar, and both scored high on competitiveness; however, Paralympic athletes scored significantly higher in mastery orientation, and the authors postulated this could be the byproduct of having to negotiate and master skills in relation to their impairment (e.g., adjusting to the use of wheelchair, learning to cope with unexpected barriers such as staircases). Understanding stable functional constraints such as personality traits and tendencies could help shape skill development to push athletes to their limits while keeping athletes engaged. Furthermore, it is important to consider subtle individual differences and how each may respond differently to certain task demands (Pinder and Renshaw, 2019).

#### Malleable Functional Constraints

Internal factors including psychological qualities such as fear, mood, and self-efficacy also affect development and performance (Glazier and Robins, 2013). Many functional constraints have a rapid rate of change, which makes them more variable over time and more malleable. For example, individuals with recently acquired impairment are prone to lower self-efficacy and lower motivation to participate in sports (Greguol et al., 2015; Veldhuijzen van Zanten et al., 2015). Ironically, there are numerous reports on the benefits of sport participation on participants’ self-efficacy (Martin, 2013; Perrier et al., 2015), although the dose-response relationship appears to depend on the sport (i.e., wheelchair sports with more dynamic and unpredictable movements result in higher self-efficacy than less dynamic wheelchair sports and/or non-wheelchair sports; Fliess-Douer et al., 2003). In addition, athletes with more experience in sport display a different type of anxiety profile and have less pre-competition state anxiety (Ferreira et al., 2007) as the extensive experience in competitive climates can help mitigate the initial “participation butterflies” (Gioia et al., 2006). Furthermore, in an optimal participation environment, numerous benefits have been reported, such as increased sense of accomplishment, decreased anxiety and depression, enhanced mood, higher self-efficacy, better general competence, as well as enhanced object control, locomotor skills, and self-perception (Jefferies et al., 2012; Martin, 2013; Martin and Malone, 2013; Martin and Vitali, 2014). Interventions such as mindfulness have also had a positive impact on athletes’ psychological flexibility and perceived stress (Lundqvist et al., 2018).

Therefore, using the constraint-led approach to design practice sessions could maximize athletes’ current action capabilities which can improve psychological qualities such as motivation and self-esteem, which will “feed-forward” into future positive behaviors. On the one hand, the ability for rapid change in malleable functional constraints makes them important for coaches, trainers, and administrators working in Para sport contexts. On the other hand, however, malleable functional constraints may limit opportunities for development/participation if an individual’s motivation to begin or maintain involvement is contingent on the availability of appropriate environments.

#### Unstable Functional Constraints

While malleable constraints are unstable and adaptable by nature, unstable functional constraints are more transient and emphasize the day-to-day variations that impact athlete training and performance. Unlike long-term psychological factors that impact and are impacted by sport participation, an athlete’s daily mood can be influenced by a wide range of factors including elements within sport (e.g., recent dialog/interaction with coaches, other athletes) and outside of sport (e.g., family and friends, work-related factors). One’s mood and state of emotion can impact their visual perception, visual field, anticipated action, and information that can be readily and immediately used for cognitive processing (Zadra and Clore, 2011). Emotional arousal can also enhance the learning process (Wolfe, 2006; Hu et al., 2007). Thus, one’s current emotion and mood may be a mediator to identifying important environmental cues in the learning and execution of tasks. Coaches and practitioners would benefit from being mindful of this variability between sessions and how it may impact athlete behavior and performance and ultimately task outcome.

### Performer Constraints Summary

Given the complexity and a wide range of factors that impact an individual’s system and behavior, the ability to recognize each factor and its origin is important. For example, the ability to differentiate between factors that may be malleable or unstable could influence coaching philosophy in practice. In a testing environment, malleable factors must be considered as day-to-day and controlled for variability between testing sessions. In a practice context, a coach’s awareness of this may allow them to be more lenient toward negative consequences associated with malleable factors and less forgiving in situations where unstable traits are present. Therefore, a case-by-case approach is ideal as the response to each circumstance will depend on the nature of the issue and a deeper understanding of constraints behaviors can mediate how one approaches each scenario.

### Task Constraints

In Newell’s original model, task constraints were categorized under one category; generally, task constraints relate to the requirements of the sport such as physical demands (e.g., strength, aerobic vs. anaerobic energy systems), the rules, parameters (e.g., court dimensions, playing surface, equipment), and the different roles within the sport (e.g., positional demands). However, due to the inherent complexity of Para sport and a need for a framework that better addresses these complexities, using our expertise, we have organized the current literature in task constraints by introducing four new sub-categories (“general,” “outcome,” “sport-specific,” and “skill-specific”) that better contextualize the role of “task” in this dynamic relationship.

#### General Task Constraints

Within the scope of our discussion, general tasks are the primary factors within each sport, such as the ability to push and control one’s wheelchair, grab and control a racquet (e.g., table-tennis, tennis, badminton), control a stick with the mouth (i.e., boccia), sit/stand on skis, and so on. While general task constraints also constitute factors necessary to operate daily activities (e.g., going up and down a ramp, getting out of bed, cooking through manipulating a fork or spatula), it is beyond the scope of this article to cover the extensive research that has been accumulated within this topic. Nevertheless, the basic task requirements are essential for sport entry which highlights the importance of a strong foundation at the grassroots level. Athletes’ mastery of general skills may be vital to remaining in sport and it is noteworthy to highlight the importance of sport and physical activity as methods for recovery and adjustment to impairment for individuals with newly acquired injuries (Murphy et al., 2008; Day, 2013; Bourke et al., 2015; Martin-Ginis et al., 2016).

#### Outcome Task Constraints

This sub-category of task constraint focuses on outcome measures in sport-specific contexts, measured either as the outcome of the game or specific task (e.g., rebounds, points, volleys returned). Most outcome measures are assessed in association with other constraints. For example, the relationship between outcome task constraints and structural constraints has been reported to be an important predictor for trunk stability in wheelchair basketball which in turn mediates specific roles on-court (Vanlandewijck et al., 2003). In addition, athletes’ ability to cover more distance on the court in wheelchair rugby has a strong relationship with athletes’ VO2 max which is moderated by the nature and severity of impairment (Goosey-Tolfrey and Leicht, 2013), which also impacts participant roles and tactics. In team sports such as wheelchair basketball, a point system limits numbers of athletes on the court at the same time and the basis for this classification system is athletes’ functional mobility. Therefore, there are possible tactical advantages if an athlete can outperform baseline expectations in their classification. Continuing to monitor and maximize the interaction among various task and outcome constraints may result in tactical advantages coaches can utilize during recruitment and development.

#### Sport-Specific Task Constraints

Sport-specific constraints are rules, parameters, and equipment within the sport that provides competitive structure. This category of constraints has an important impact on Para sport athletes’ development both in theory and practice. For example, the varied health experiences of these athletes underscore the challenges associated with meeting unique sport and task demands. Importantly, it may be a mischaracterization to view these constraints solely as limiting; certain task constraints may also act as “enabling” factors to Para sport participation and performance. For example, impairment classification is a central characteristic of Para sport and presents arguably the most obvious task constraint. Classifications reflect the International Paralympic Committee’s (IPC) objective “to support and co-ordinate the ongoing development of accurate, reliable, consistent and credible sport-focused classification systems and their implementation” (IPC, 2016) and there has been a surge for an evidence-based classification system considering the taxonomy, validity, reliability, and unification of testing across each sport (Tweedy, 2002; Beckman and Tweedy, 2009; Vanlandewijck et al., 2011; Beckman et al., 2017; Tweedy et al., 2018). More specifically, classification is based on an athlete’s physical ability and capability to perform sporting tasks, although there is evidence suggesting that disability severity may not, in fact, be a significant indicator of *potential* to reach expertise (Hedrick et al., 1988). Dehghansai et al. (2017a,b) supported this notion through recent examinations of Para sport athlete development, reporting that disability severity may not influence athletes’ progression to elite status, despite an overall lack of research on sport-specific development of Para sport athletes. However, disability severity has the potential to negatively affect Para sport athlete *selection* and subsequent development (i.e., tasked to implement a set of tactical behaviors which prevents the athlete to develop a wide range of skills within the sport).

As such, classifications can be viewed from both an exclusionary and inclusionary perspective. For example, as classifications are sport-specific and relative to the unique demands of each sport and a broad spectrum of individual limitations, some athletes may meet the criteria to participate in one sport, but not another (Baker et al., 2017). Furthermore, Para sport practitioners may altogether bypass individuals with specific impairments that negatively affect their ability to perform a sport-related task (perceived or actual), such severity of athlete’s SCI affecting their trunk movement and ability to rebound the ball in wheelchair basketball. On the other hand, classifications may also be viewed as a means to facilitate participation in appropriate sports regardless of the athletes’ intrinsic motivation for that particular sport. Therefore, a task constraint such as impairment classification may limit *or* help shape skill attainment (i.e., skill-specific task constraint) and development ultimately leading to a successful or failed outcome (i.e., outcome task constraint). This highlights the symbiotic nature of these sub-categories and the need to consider the dynamic interactions occurring among various factors.

#### Skill-Specific Task Constraints

Skill-specific constraints refer to the individual’s ability to adopt and excel in a specific task (e.g., the forehand in wheelchair tennis). While many sport interactions consist of a dynamic interplay between two athletes, the focus of this constraint is on specific tasks within the game. More specifically, the attention is shifted to develop a better understanding of task manipulation and execution, and the underlying mechanism of how a specific skill is learnt and performed (e.g., the rolling build-up and execution of a lay-up in wheelchair basketball). From the very limited literature on this topic in Para sport, an individualized approach is suggested as each athlete approaches learning and responds to various task manipulations within practice differently (Pinder and Renshaw, 2019). The individualized approach utilizing task manipulation for acquisition and modification of specific skills has reportedly had a positive impact on athlete’s ability to acquire and transfer learnt skills into different performance contexts (Pinder and Renshaw, 2019).

### Task Constraints Summary

Differentiation between the type of task constraints and a deeper understanding of the fundamentals that create the dynamic complexity of a game scenario could be extremely helpful to coaches and practitioners. While it is important to understand the layers that construct the execution of a movement, a holistic approach that considers the interaction of these complex movements is equally important. As seen within this extended framework, individual constraints directly interact with task constraints and shape behavior. Therefore, when designing tasks and considering session outcomes, conceptualizing and understanding the behavior of microelements of a complex task provides a deeper perspective on the collection of behaviors that shape the performance of an athlete.

### Environmental Constraints

Environmental constraints are less stable (i.e., more dynamic) influences that do not change the goal of the skill and/or sport-specific task, but can influence development and performance. While the original description of this constraint in Newell’s (Newell, 1986) model contained no sub-categories, we feel that it is important to provide more nuance given the complexity of athlete development in general, and particularly in Para sport. The proposed sub-categories below, natural, infrastructure, sociocultural, and interpersonal, are consistent with modern ecological systems theories (Bronfenbrenner, 2005; Bronfenbrenner and Morris, 2006; Gottlieb et al., 2006; Spencer, 2006), and emphasize the importance of incorporating different layers of the ecology into theoretical frameworks and the design of applied learning environments. This highlights the complex interaction across multiple variables that can impact individual’s development from one’s immediate environment (micro) to the larger community (meso) and cultural/historical aspect of the society (Bronfenbrenner, 2005).

#### Natural Environmental Constraints

Natural environmental constraints (e.g., climate and geographic position) can influence athletes’ sport selection, development, and overall performance. For example, training year-round for winter sports is extremely difficult in southern countries such as Brazil, where the climate itself can hinder/enhance athletes’ development and performance. Training in cooler temperatures and competing in contrasting climates (e.g., Canadian athletes competing in the recent Rio Paralympic Games) can impact an athletes’ performance. In addition, training in hotter and humid climates is difficult for athletes with impairments affecting their autonomic nervous system and thermoregulation (Mills and Krassioukov, 2011). Griggs et al. (2015) reported that tetraplegic athletes had a higher body temperature than paraplegic athletes during the same workouts. Therefore, it is exceedingly important to consider athletes’ specific impairments and their interaction with training and competition contexts. In turn, this may mitigate negative biopsychosocial outcomes and improve athletes’ training and performance (i.e., influencing skill-specific, sport-specific, and outcome task constraints).

In addition, athletes’ physical location can mediate the distance traveled to practice (e.g., a country with a greater surface area such as Canada vs. smaller surface area such as Germany). Living in a country with a greater surface area can result in higher costs of transportation, difficulties in planning transportation, and longer commutes (Radtke and Doll-Tepper, 2014). The daily commute to training facilities has been one of the predominant barriers Para sport athletes face (Martin and Whalen, 2014). Due to the distance between team members, athletes and coaches rely on training camps and electronic methods of communication to build relationships and develop team chemistry (Falcão et al., 2015). Lack of teammates and support networks has been a reported issue for athletes residing in remote areas (Kean et al., 2017). Interestingly, centralized training environments have been reported to positively contribute to athletes’ training quality, provide a platform for necessary feedback, and enable additional social support while providing national sporting organizations an opportunity to channel funding and evaluate program designs more effectively (Kean et al., 2017). In addition, athletes’ geographic location can impact their ability for sport classification. Therefore, coaches and practitioners may turn to e-communication to compensate for time away from the team in order to maintain chemistry and if funding allows, facilitate regularly centralized camps to build rapport, provide up-to-date relevant feedback, and enhance the quality of athletes’ training.

#### Infrastructure Environmental Constraints

Infrastructure environmental constraints directly hinder or facilitate development and performance through how they affect availability, accessibility, and/or affordability (Mwangi et al., 2009; Goodridge et al., 2015; Kean et al., 2017). Availability and accessibility to training facilities (e.g., curb cuts, location of change rooms/bathrooms) has been a long-standing barrier for Para sport athletes (Jaarsma et al., 2014; Martin and Whalen, 2014; Martin-Ginis et al., 2016). The limited infrastructure reduces flexibility for a number of programs available which are bound to specific times of the day and require a certain number of regular participants. Considering participation rates across communities are already reported to be low, programs with limited attendees can be eventually removed, cyclically reducing the number of programs further and negatively affecting one of the leading sport participation barriers (Stephens et al., 2012; Martin-Ginis et al., 2016). The limited opportunities within the community, in turn, negatively affect individuals’ motivation to participate (i.e., a functional malleable constraint). In addition, training outdoors has its own limitations including road safety and security. Therefore, from a coaching standpoint, ensuring the facility is accessible, the training environment is barrier-free and athletes can navigate to meet task demands can be vital to athletes’ attitude toward participation. From a policy standpoint, understanding these limitations can have a systematic impact on the growth of the Games: removing barriers may increase participants which in turn can contribute to the pool of athletes that compete across the pathway.

#### Sociocultural Environmental Constraints

These constraints encompass higher-order factors (i.e., policies, laws, social beliefs, and attitudes) that can indirectly impact individuals’ development and their surrounding social structure. The “Accessibility for Ontarians with Disabilities Act,” established in 2005, is an example of a Canadian government policy targeting barrier removal for individuals with impairments. The aim of this act is to ensure all facilities are accessible to individuals with impairments (Accessibility laws, 2017). However, without agencies implementing and enforcing this Act, its existence alone does not seem to have had a comprehensive impact on the accessibility of infrastructures (Martin-Ginis et al., 2016).

Recent reports examining the distribution of funding across agencies highlights concerns associated with programs at the regional and provincial levels with the majority of funding transmitted to the national and international level of sporting organizations (Radtke and Doll-Tepper, 2014). A recent comprehensive review exploring sport participation barriers among individuals with impairments reported that policies have had a direct impact by influencing available funding opportunities to increase program availability, transportation services, and staff education, which coincidentally are frequently reported barriers to sport participation (Martin-Ginis et al., 2016). In conjunction with unbalanced funding, the lack of communication between the hierarchies can have detrimental effects on individuals interested in participation all the way from grassroots to competitive levels.

#### Interpersonal Environmental Constraints

Interpersonal environmental constraints include the various influential agents and social support systems in a performer’s life, such as coaches, teammates, doctors, parents, and friends. The importance of family and coach support (e.g., emotional, financial) in successful athlete development has been extensively investigated (Bloom, 1985; Côté, 1999), and while strong family support is common in Para sport, (Medland and Ellis-Hill, 2008) there is a lack of specialist coaches (Martin, 2015). Historically, coaching in Para sport has been a challenge (Townsend et al., 2016); while some athletes have learned to coach themselves, others have been coached by individuals whose primary involvement (either as a coach and/or athlete) has been in able-bodied sports (Duarte et al., 2018). Coaches also find it challenging to locate information on sport-specific training and disability (Hammond et al., 2014; Vargas et al., 2015), and some coaches seek help from parents to develop a better understanding of athlete’s unique needs and action capabilities (Martin, 2014). Lack of coach development and education programs lead to coaches relying on experiential knowledge and informal communication with colleagues as means of coach development (Dehghansai et al., 2019), even reaching out to coaches from different sports (Duarte et al., 2018). In addition, recent evidence has found that Para sport coaches tend to progress through the coaching ranks to national coaching positions relatively quickly and may not be fully equipped with the necessary experience, knowledge, or resources to support their transition (McMaster et al., 2012; Douglas et al., 2018). Therefore, new coaches could benefit from networking with other coaches in Para sport (including attending conferences, workshops, etc.), while more experienced coaches can contribute to the Para sport system by serving as mentors and extending their experiential knowledge to newer coaches. It may also help coaches (especially at earlier stages of athletes’ career) to maintain clear communication with parents and caregivers to better understand the unique considerations necessary for each athlete. Utilizing the extended framework as a guide and devising training sessions considering the constraint-led approach can benefit both experienced and novice coaches (see Pinder and Renshaw, 2019 for how this framework has been utilized in practice). In this, short- and long-term plans can be considered while taking into consideration the influence and support of the individuals within athletes’ social system.

### Environmental Constraints Summary

As highlighted previously in the literature, understanding the complex relationship between different layers of the environment requires extensive examination of the foundational elements that make up these layers. A more concerning issue is the impact indirect factors (e.g., policies, historical or societal views) can have on athletes’ experience and development. Therefore, it is important to continue to develop a deeper understanding of the complex relationship between environmental factors and their influence on development and performance. The challenge for coaches becomes the interaction of the deeply rooted systematic problems (i.e., policies, infrastructure) and their impact on athletes’ experience. While, in some scenarios, a coach may have the ability to influence a policy change and alteration of an infrastructural barrier, at other times, it may be just as important to be aware of these challenges to educate and inform parents, caregivers, and athletes of the nuances associated with the sporting experience.

## Discussion

Newell’s model and the suggested extended categories noted above reflect numerous factors that influence the development of Para athletes, highlighting the need to utilize frameworks that identify and acknowledge the complexities associated with athlete development. With this in mind, coaches can benefit from the understanding of this complex interaction between the myriad factors that influence athlete development at any given time, and this extended framework allows coaches and practitioners to consider these complexities when designing optimal performance environments. These factors should be considered across time, from micro- (immediate, on-ground daily training environments) to macro-levels (long-term training programs, policies, and resource allocation) to better organize and structure athletes’ development.

It is vital to approach athletes’ development from a holistic standpoint. Utilizing the three overarching constraints of Newell’s theoretical framework and organizing developmental factors within each sub-category, coaches can capture and understand the dynamic and complex interaction between variables that contribute to athletes’ experience and development. For example, recommendations for appropriate training and sport-specific guidelines can vary between athletes due to disability-related factors, athletes’ biological age, and sport-readiness; as a result, training tasks or methodologies may work for one group of athletes but not others. In addition, there may be limited resources available to implement ideal training routines, therefore requiring further modifications for practicality reasons. Another solution may be to locate a new facility that has the necessary equipment, but coaches need to be cognizant of accessibility (infrastructure and transportation access). Therefore, in addition to considering individual-related factors (e.g., nature and severity of impairment, previous sporting experience), it is vital to consider the interaction of social factors such as family dynamics, social networks, and infrastructures and their impact on athlete development. While the constraint-led approach can provide great benefits for athletes, as it is tailored uniquely to their specific needs, it does present challenges for coaches. For instance, each athlete needs to be assessed independently which may increase the workload for coaches. In addition, first attempts can be overwhelming due to the complexity of the interactions and the multitude of factors to consider. We recommend starting with small, controllable tasks and slowly progressing to more complex environments. These smaller pieces will slowly integrate and emerge into a complete picture. This picture will evolve but through familiarity and trial and error, we believe coaches can become comfortable with the uncertainties that are presented and learn to prepare for the range of expected and unexpected events that are presented across their athletes’ developments (see Pinder and Renshaw, 2019, for an application of this approach in the Para sport context).

The factors mentioned above are just a small sample of factors that interact and impact athletes’ development. Therefore, devising a recommendation guideline or athlete development model for coaches is beyond the scope of this paper. Rather, our intention has been to provide a framework to guide the coaches’ planning. In turn, this extended framework can help coaches devise and better plan for demands that may be present in the process of athletes’ development. The preparedness can better equip coaches to deal with these events, ultimately leading to a more efficacious learning environment for the athlete.

## Conclusion

Our primary goal in this discussion paper was to frame current literature using Newell’s framework and provide additional categories to guide coaches’ planning and preparation for their athletes’ development. There are complex interactions between factors associated with athlete development and this dynamic synergy is further complicated by the unique influences of different impairments in Para sport contexts. A holistic approach that considers the interaction between an athlete’s proximal environment and indirect societal factors may provide a better overview of optimal developmental trajectories. Further, sport-specific considerations must include the dynamic interplay of impairment differences among athletes within training environments. While adapted models from able-bodied sports try to compensate for the current shortcomings in the Para sport development literature, the performers and coaches who make up this population need and deserve considerations that better reflect the unique constraints affecting the development of high-performance Para sport athletes.

## Author Contributions

All authors were in charge of a section in the manuscript, reviewed the manuscript, and provided feedback. ND was responsible to collate all the sections and prepare the manuscript for submission and also ensured formatting and manuscript alignment according to the journal’s guidelines.

## Conflict of Interest

The authors declare that the research was conducted in the absence of any commercial or financial relationships that could be construed as a potential conflict of interest.

## Footnotes

1 It is important to note that in Newell’s model constraints both impede and facilitate outcomes.
